# Defining the role of the tumor vasculature in antitumor immunity and immunotherapy

**DOI:** 10.1038/s41419-017-0061-0

**Published:** 2018-01-25

**Authors:** Marco B. Schaaf, Abhishek D. Garg, Patrizia Agostinis

**Affiliations:** Cell Death Research & Therapy (CDRT) Laboratory, Department for Cellular and Molecular Medicine, KU Leuven University of Leuven, Leuven, Belgium

## Abstract

It is now well established that cancer cells co-exist within a complex environment with stromal cells and depend for their growth and dissemination on tight and plastic interactions with components of the tumor microenvironment (TME). Cancer cells incite the formation of new blood and lymphatic vessels from preexisting vessels to cope with their high nutrient/oxygen demand and favor tumor outgrowth. Research over the past decades has highlighted the crucial role played by tumor-associated blood and lymphatic vasculature in supporting immunoevasion and in subverting T-cell-mediated immunosurveillance, which are the main hallmarks of cancers. The structurally and functionally aberrant tumor vasculature contributes to the protumorigenic and immunosuppressive TME by maintaining a cancer cell’s permissive environment characterized by hypoxia, acidosis, and high interstitial pressure, while simultaneously generating a physical barrier to T cells' infiltration. Recent research moreover has shown that blood endothelial cells forming the tumor vessels can actively suppress the recruitment, adhesion, and activity of T cells. Likewise, during tumorigenesis the lymphatic vasculature undergoes dramatic remodeling that facilitates metastatic spreading of cancer cells and immunosuppression. Beyond carcinogenesis, the erratic tumor vasculature has been recently implicated in mechanisms of therapy resistance, including those limiting the efficacy of clinically approved immunotherapies, such as immune checkpoint blockers and adoptive T-cell transfer. In this review, we discuss emerging evidence highlighting the major role played by tumor-associated blood and lymphatic vasculature in thwarting immunosurveillance mechanisms and antitumor immunity. Moreover, we also discuss novel therapeutic approaches targeting the tumor vasculature and their potential to help overcoming immunotherapy resistance.

## Facts

- Cancer cell and stromal cell interface enforces a tumor microenvironment (TME) that is permissive for tumor growth.
- The dynamic properties of the TME regulate how malignant cells respond to therapy.
- Cancer cell-derived proangiogenic factors triggers unproductive angiogenesis and lymphangiogenesis that facilitate tumor growth and metastasis.
- The structurally and functionally abnormal tumor blood and lymphatic vasculature favor escape of malignant cells from antitumor immunity and fosters the immunosuppressive TME.
- Endothelial cells (ECs) of the tumor vasculature actively suppress antitumor immunity by regulating recruitment, adhesion, and function of immune cells and by inducing killing of effector T cells.
- A complex bidirectional interface between tumor vasculature and the immune cells regulates therapy responses.
- Targeting the tumor vasculature with antiangiogenic agents allows a transient improvement of the vessels that improves tumor oxygenation and enhances drug delivery, immune cells' infiltration, and immunotherapy efficacy.

## Open questions

- What are the molecular mechanisms regulating the intense crosstalk between ECs and immune cells within the TME?
- What is the role of other stromal cells (e.g., cancer-derived fibroblasts) in tumor angiogenesis?
- Which vasculature-targeting approaches can ‘heat up’ the TME and favor infiltration of T cells?
- Which tumor vasculature-targeting regimens create the best window of opportunity required for a durable effect on immunostimulating TME?
- Which pathway and EC-specific molecular target should we target to improve therapy responses?
- How should the lymphatic system be targeted considering that it serves peripheral tolerance but also facilitates adaptive immune response by draining tumor-associated antigen(-presenting DC)?
- What are the best treatment scheduling options for antiangiogenic therapies when combined with immunotherapy modalities?
- Do tumor vessel-normalizing strategies offer a best treatment strategy to improve T-cell function and immunotherapy?
- Does the concept of vessel normalization extend to the lymphatic vasculature and what are the underlying mechanisms?
- Do vessel-normalizing strategy in combination with immunogenic cell death-based approaches synergize?
- Which biomarkers will allow monitoring the effects of vessel normalizing drugs on patient’s immunological responses to therapy?

## The crosstalk between cancer cells and stromal cells shapes the tumor microenvironment

In recent years, tumors have been recognized as complex dysorganized and chaotic organs, where cancer cells co-exist and co-evolve with their stroma. This view is a major shift from the previously accepted ‘cancer cell-centered’ perception of cancer evolution, which mainly focused on understanding oncogenic drivers and cell-autonomous features of cancer. It is now increasingly accepted that the interface between malignant and non-transformed cells defining the tumor microenvironment (TME), represents a highly plastic tumor ecosystem that supports tumor growth and dissemination through the various stages of carcinogenesis. Apart from cancer cells, the TME of a solid tumor contains a complex interstitial extracellular matrix and various stromal cells that are recruited from the surrounding tissues or from the bone marrow^1^ and include fibroblasts, cells of the immune systems, pericytes, and ECs of the blood and lymphatic vasculature.

Within the TME, cancer cells thrive and maintain a dynamic communication with all TME components through the release of soluble factors (e.g., cytokines, chemokines, growth and inflammatory factors, lipid mediators, matrix remodeling enzymes) or through cancer cell–stromal cell contacts, which ultimately drive a chronic inflammatory, immunosuppressive, and pro-angiogenic niche that promotes dissemination of cancer cells and thwarts the effects of various therapeutic interventions, including immunotherapy. Moreover, this intersection is bi-directional, since each stromal component of the TME may establish a proficient interface with cancer cells, which facilitates cancer progression, at virtually any stage of tumorigenesis.

For example, a large body of experimental evidence supports the concept that the immune system is able to eradicate emerging tumors through the process of cancer immunosurveillance before cancer cells evolve the ability to erode detection and eradication by immune cells^2,3^. Distinguishing mechanisms enabling an immunoevasive cancer cell phenotype include a reduced immunogenicity due to loss in expression of tumor-associated antigens (TAAs) or major histocompatibility complex (MHC) class I molecules, acquired DNA copy number alterations and oncogenic signaling, upregulation of cellular immune checkpoints like programmed death ligand 1 (PD-L1), indoleamine 2,3-dioxygenase (IDO), and tryptophan 2,3-dioxygenase (TDO), and altered metabolism resulting in a low pH and secretion of various metabolites^4^. Moreover, through increased production of immunosuppressive and tumor-promoting cytokines, cancer cells modulate the polarization, activity, and expansion of various immune cell subpopulations and interface with ECs, causing alterations in their structural integrity and functional properties, thus diminishing antitumor immune responses.

In fact, cancer immunosurveillance, which is driven largely by activated effector T cells, is impaired at different levels by several obstacles imposed by the increasingly hostile TME. To exert their function, T cells need to be properly activated by antigen-presenting dendritic cells (DCs), usually by encountering DCs in peripheral lymph nodes (LNs), egress the LNs and home to the tumor and finally extravasate from blood vessels and infiltrate the tumors. Thereafter, activated CD8^+^ T cells can recognize TAA presented through a MHC class I molecule on cancer cells and induce their killing via the perforin-granzyme and/or Fas ligand (FasL)/tumor necrosis factor (TNF)-related apoptosis-inducing ligand (TRAIL) systems, although this depends on the degree of functional inhibition by the TME and the presence of immunosuppressive regulatory T cells (T_regs_), myeloid-derived suppressor cells (MDSCs), and tumor-associated macrophages (TAMs). In this scenario, the blood and lymphatic vasculature have important roles as physical and functional barriers for tumor-infiltrating immune cells and TAA/TAA-presenting DC drainage to the LNs, respectively.

Finally, such an intense crosstalk between cancer cell and stromal cells not only promotes tumor growth and dissemination but also gravely affects the efficacy of multimodal anticancer treatment. This is particularly true for the currently, clinically used cancer immunotherapies, such as those employing immune checkpoint inhibitors (ICIs) or adoptive T-cell transfer (ACT), that primarily aim to reinvigorate antitumor T-cell activity.

Here we aim to discuss the current view on the cancer cell-induced alterations in the blood and lymphatic vasculature as well as (sentinel) LNs that profoundly impede antitumor immunity. We also summarize the advances and therapeutic combinations targeting the tumor vasculature that may overcome immunotherapy resistance.

## Tumor-associated blood vasculature favors an immunoresistant tumor microenvironment

Solid tumors that have grown beyond few cubic millimeters need to induce tumor angiogenesis, to receive nutrients (e.g. oxygen and glucose) required for their high energy demand and growth. Tumor angiogenesis entails the development of new blood vessels from established vascular beds and as such is different from vasculogenesis (de novo formation of vessels from bone marrow-derived endothelial precursor cells) or vasculogenic mimicry (the ability of tumor (stem) cells to form vessel-like networks)^5^. The formation of a novel vascular sprout is a dynamic and tightly orchestrated process that involves the coordinated action of the highly invasive and motile tip cells at the leading edge (migrating towards pro-angiogenic cues and guided by key pro-angiogenic vascular endothelial growth factor (VEGF)-A–vascular endothelial growth factor receptor-2 (VEGFR-2) axis) and the underlying proliferating stalk cells, elongating the sprout and generating the lumen. Such fully formed vessel recruits pericytes and vascular smooth muscle cells thereby promoting stability, integrity, and blood perfusion (extensively reviewed in refs. 6,7).

**Fig. 1:**
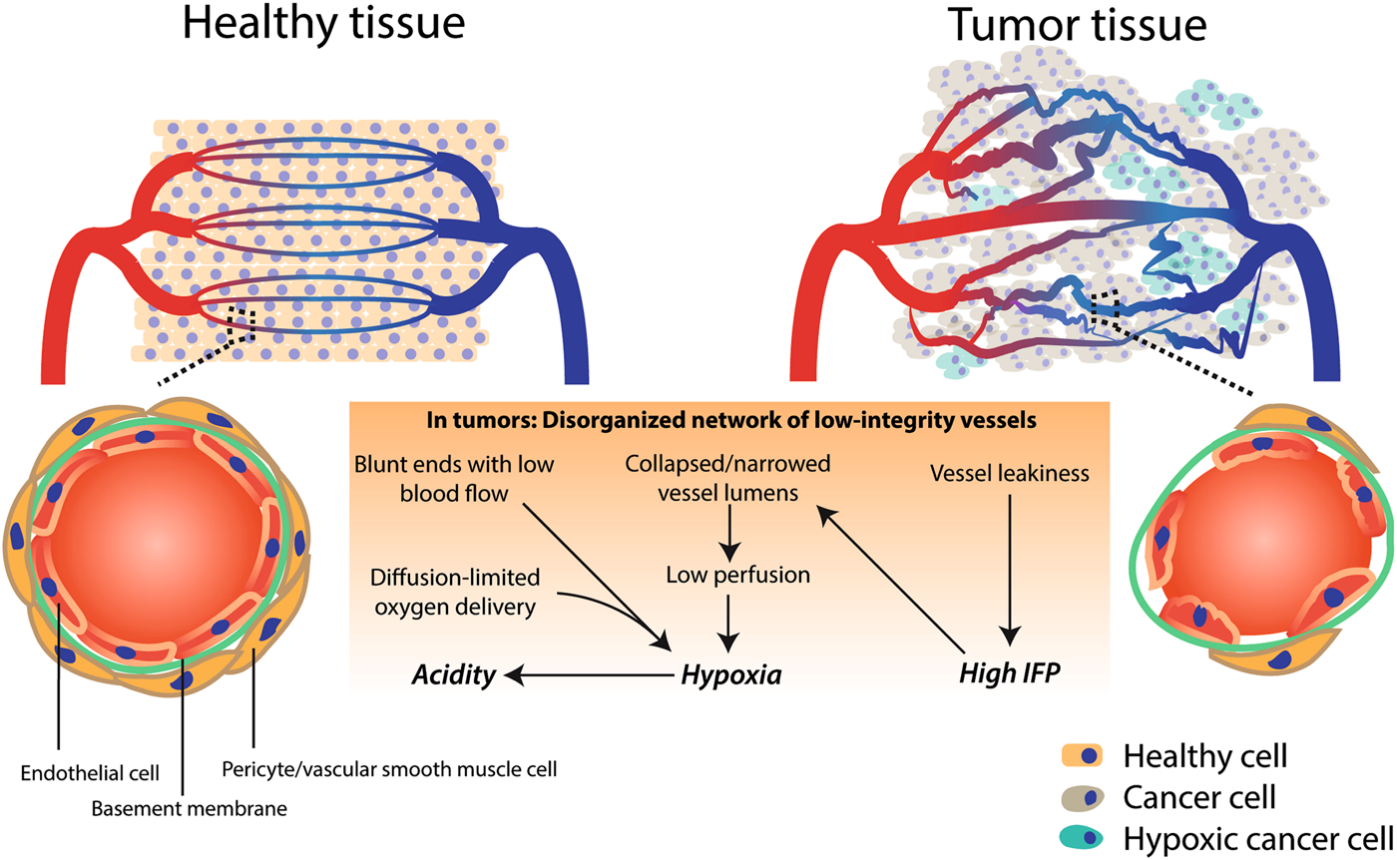
**Tumor-associated blood vasculature is a major influencer of the tumor microenvironment (TME)** (Upper left panel) A well-organized vessel network ensures full-covering of nutrient supply. (Lower left panel) These vessels are matured with an endothelial cell layer surrounded by a basement membrane and pericytes (like smooth muscle cells). The endothelial layer is characterized by tight intercellular junctions. Oppositely, due to high pro-angiogenic signaling, the network of tumor-associated blood vessels (upper right panel) is chaotic, low in pericyte coverage and has loose inter-endothelial cell junctions (lower right panel). This generates leaky vessels that increases interstitial fluid (IFP) pressure. Common blunt-ended or collapsed vessels results in tumor regions that are starved from nutrients including oxygen (hypoxic cells indicated in green). Moreover, the glycolytic nature of the (hypoxic) tumor cell acidifies the pH in the TME

Pathological angiogenesis is mainly driven by an imbalance between pro-angiogenic and antiangiogenic signaling in the TME. Key pro-angiogenic factors include, but are not limited to, VEGF-A, basic fibroblast growth factor (bFGF) and interleukin (IL)-8. These cytokines become ubiquitously abundant in the TME and overwhelm angiostatic signals, such as angiostatin and endostatin, thereby inducing a pro-angiogenic switch^8^. In fact, not only cancer cells secrete high amount of VEGF and can contribute to VEGF-independent angiogenesis (by liberating various pro-angiogenic molecules, such as placental growth factor (PlGF), VEGF-C, VEGF-D, and platelet-derived growth factor (PDGF)-C) but they can also respond in an autocrine or paracrine manner to prosurvival and prometastatic VEGF signaling^5^. Although tumor angiogenesis is meant to support blood supply to the tumor, the resulting vessel network is leaky, chaotically organized, immature, thin-walled, and ill-perfused (Fig. 1). This unproductive, highly aberrant angiogenesis contributes to maintain the protumorigenic and immunosuppressive TME and profoundly influences how cancer cells escape the anticancer immunosurveillance, metastasize, and respond to immunotherapy.

A chaotic vascular network, which gives rise to blunt-ended vessels and inconsistent blood flow^9^, is associated with structurally immature vessels that are unstable, leaky, and tortuous. Ill-covered perfusion results in diffusion-limited nutrient delivery (cells are too far from functional vessel). Moreover, due to the high interstitial fluid pressure (IFP) in the tumor (a result of vessel leakiness) these vessels are prone to collapse and diminish the perfused tumor area (Fig. 1)^6^.

This generates a hypoxic (i.e., less oxygenated) and acidic (due to increased anaerobic glycolysis of cancer cells) TME that facilitates the selection of cancer cells with genetic (i.e., enumeration of mutations favoring malignancy) and epigenetic alterations that enhance their aggressiveness. Importantly, hypoxia and acidosis (reviewed in ref. 10) facilitate attraction/development of immunosuppressive immune cells, reduce the cytotoxic activity of tumor-infiltrating effector T cells, and hamper delivery of chemotherapeutics and immunotherapeutic entities, as well as cancer cell killing in response to radio/chemotherapy and immunotherapy (as discussed later).

**Fig. 2:**
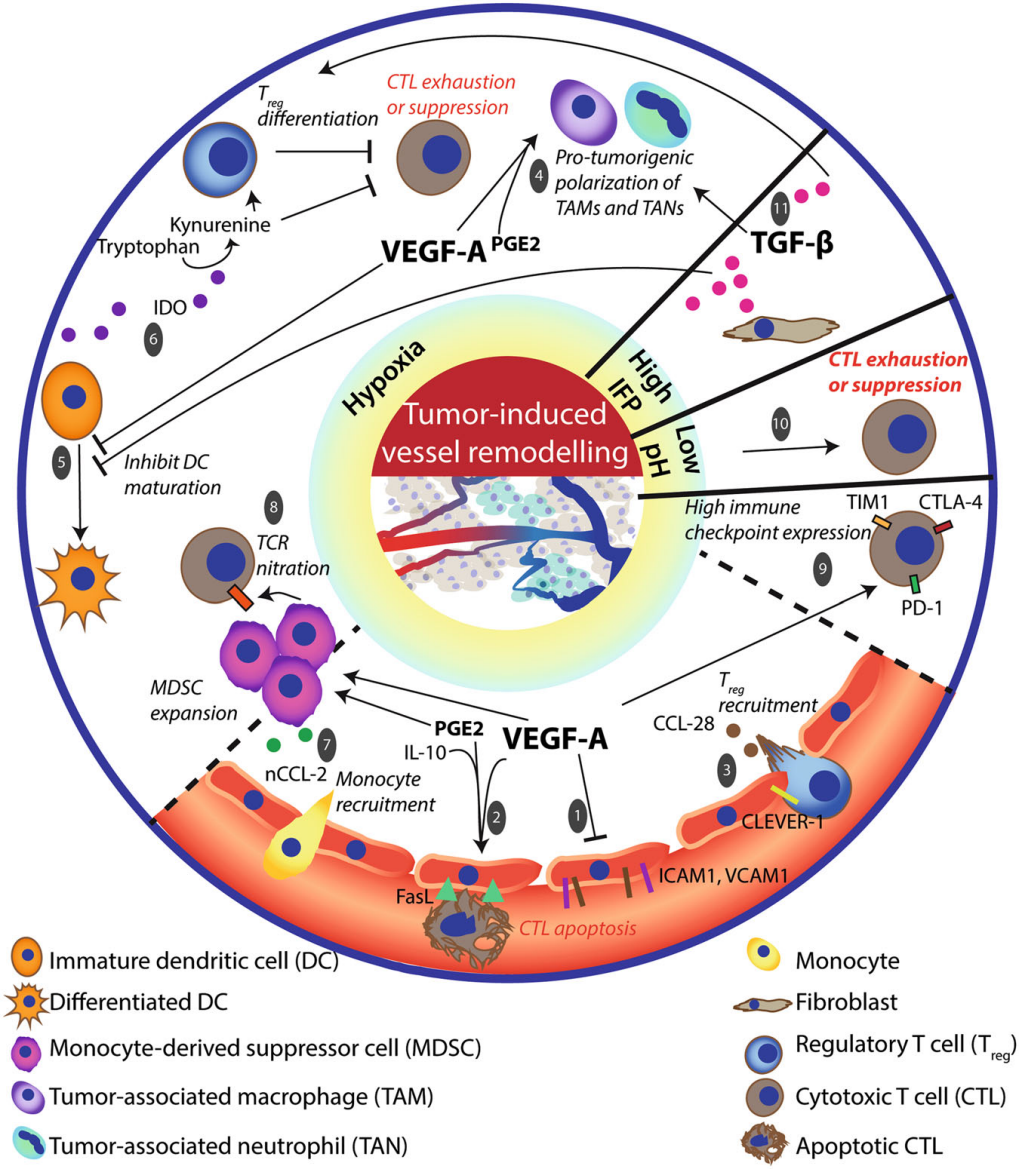
**The nature of the TME influences immune cell composition and hampers antitumor immunity** First, hypoxia is a common feature of the TME caused by the abnormal vascular structure and function. Dysregulated adhesion [1] and differential admittance among immune cell types is caused by several hypoxia-related factors in the TME, including VEGF-A, PGE_2_ and IL-10. Together these induce FasL expression on ECs that affects survival of effector T cells (rather than T_regs_). [2] In addition, expression of CLEVER-1/stabilin-1 on tumor-ECs and hypoxia-related chemokine CCL-28 in the TME further aid the recruitment of, preferentially, T_regs_. [3] The hypoxic TME recruits monocytes that give rise to MDSC, TAM, and TAN populations [4] in the tumor and induces a differential and functional immature phenotype of DCs. [5] This collectively supports an immunosuppressive TME. Immature DCs produce IDO to favor T_reg_ differentiation from naive T cells and inhibit CTL function. [6] MDSCs are a source of reactive nitrogen species that nitrate CCL-2 and tyrosines of the T-cell receptor that recruits more monocytes [7] and impedes CTL antigen recognition, [8] respectively. Moreover, VEGF-A induces the expression of PD-1, TIM-3, and CTLA-4 on CTLs to render them more susceptible to functional inhibition. [9] Second, as a result of a more glycolytic metabolism the TME acidifies (low pH), thereby inhibiting the induction of antigen specific CTLs. [10] Third, the leaky tumor vessels induce a high interstitial fluid pressure (IFP) that leads to high TGF-β production that is also implicated in TAM M2 polarization, maintaining immature DC phenotype and differentiation and proliferation of T_regs_. [11]

Here we discuss some of the major mechanisms imposed by the erratic tumor vasculature to reverse or prevent antitumor immune responses (Fig. 2).

### Accessibility of immune cells to the tumor bed

A hypoxic TME is associated with high VEGF-A, IL-10, and prostaglandin E_2_ (PGE_2_) levels. These factors collectively induce FasL expression on tumor ECs, which upon binding to Fas expressed on T cells, triggers their killing by apoptosis. Due to a differential expression of c-FLIP (a known suppressor of TNF, FasL, and TRAIL-induced apoptosis^11^), CD8^+^ T cells are more adversely affected by these events than T_regs_^12^. Moreover, tumor-associated ECs can preferentially promote the recruitment of T_regs_ by the upregulation of the multifunctional endothelial receptor CLEVER-1/stabilin-1, thus suggesting that tumor endothelium can support both the recruitment and the survival of immunosuppressive T cells^13^. In addition, VEGF-A mediates a clustering defect of the adhesion molecules like intercellular adhesion molecule (ICAM)-1 and vascular cell adhesion protein (VCAM)-1, which hampers immune cell extravasation^14^. Thus, aside from stimulating angiogenesis, VEGF-A also contributes to the impediment of an efficient EC–lymphocyte interaction. Furthermore, the endothelin B receptor (ET-_B_R; receptor of the hypoxia-inducible factor (HIF)-1-regulated endothelin-1)) on tumor ECs is implicated in counteracting T-cell adhesion as neutralization of ET-_B_R increases tumor-infiltrated lymphocytes (TILs) and improves responsiveness to immunotherapy^15^. Although these adhesion molecules can bind multiple leukocyte subtypes, it is still unclear which compensatory signals maintain the intratumoral presence of monocytes and neutrophils.

### Maturation and polarization of immune cells

Hypoxia-induced signaling mediates the presence of certain immunosuppressive immune cell types, including immature DCs, TAMs, and tumor-associated neutrophils (TANs), as well as, MDSCs. VEGF-A is associated with reduced DC differentiation from hematopoietic progenitors^16^, and it interferes with TNF-induced nuclear factor-kB activation (important for DC functional maturation)^17^.

HIF-1 targets, VEGF-A and IL-8, aid the recruitment of immature myeloid cells that may stay undifferentiated (and develop into MDSCs) or develop into TAMs. TAMs have a high level of plasticity displaying either pro-inflammatory features (M1 phenotype) or immunosuppressive features (M2 phenotype). Hypoxia-associated molecules (e.g., VEGF-A, PGE_2_) stimulate the M2 phenotype^18^ and the expansion of monocytic (CD11b^+^) and granulocytic (Gr1^+^) MDSCs^19^. MDSCs are a source of transforming growth factor (TGF)-β, a crucial immunosuppressive factor, in the TME^20^. Importantly, these TAMs and Gr1^+^ myeloid cells can also render tumors non-responsive to VEGF/VEGFR inhibition (as mentioned later) and induce angiogenic relapse^21^.

In addition, the generated mechanical stress (due to high IFP) leads to TGF-β production from fibroblasts (reviewed in ref. 22). TGF-β promotes maintenance of immature DC phenotype that stimulates differentiation and proliferation of T_reg_ cells and thereby inhibits cytotoxic T-cell (CTL)-mediated responses^23^. Moreover, TGF-β induces IL-receptor-associated kinase (IRAK)-M expression in TAMs, important for an M2 phenotype, that has relevant implications as the growth rate of transplanted Lewis Lung carcinoma (LLC) cells was reduced in IRAK-M^−^^/−^ mice^24^. Regarding TANs, TGF-β can induce the protumorigenic N2 phenotype^25^, although it is not clear to what extent N2 cells exert long-term protumor effects since neutrophils have a relatively short life span after they leave the bone marrow and are particularly sensitive to nutrient deprivation, as typically found in tumors^26^.

### Functional activity of T-cell populations

Immature DCs may express immunosuppressive molecules, such as IDO and TDO. IDO converts the essential amino acid tryptophan in the extracellular matrix to kynurenine. Low tryptophan levels starve effector T cells while favoring T_reg_ expansion. Moreover, MDSCs are a major source of PGE_2_ that, in the absence of a pro-inflammatory milieu, tends to promote T_reg_ development, induces immunosuppressive chemokine production, and causes an increase in the barrier function of ECs by inhibiting transendothelial migration of T cells^27,28^. In addition, hypoxia-induced expression of chemokine (C-C motif) ligand-28 (CCL-28) by cancer cells recruits T_reg_^29^. The MDSCs that are attracted and expanded during hypoxic conditions can produce limited amounts of reactive nitrogen species (e.g., peroxynitrate) that can cause nitration of tyrosines in a T-cell receptor (TCR)–CD8 complex (that impedes interaction with antigen–MHC complexes^30^) and some chemokines like CCL-2. Importantly, nitrated CCL-2 can still serve as a chemoattractant for monocytes (that can function as MDSCs within a tumor) but not effector T cells^31^.

Additionally, immune checkpoints can modulate the functional activity of different T-cell populations. In this regard, the HIF-1 pathway is a major inducer of PD-L1/PD-L2 expression^32^ that inhibits the effector function of T cells (thereby inducing T-cell anergy). PD-L1/PD-L2 are commonly expressed by cancer cells, tumor-associated ECs, macrophages, fibroblasts, and DCs^33^. Moreover, VEGF-A-enhanced expression of PD-1, T-cell immunoglobulin mucin (TIM)-3, and cytotoxic T lymphocyte antigen-4 (CTLA-4) on intratumoral CD8^+^ T cells^34^ increases susceptibility to functional inhibition, thereby invigorating T-cell exhaustion. Furthermore, the acidic nature of the TME also inhibits the induction of functional CTLs from memory T cells^35^.

Collectively, these data highlight that the tumor vasculature is a crucial TME compartment with the ability to suppress both directly (e.g., through killing of immune cells) and indirectly (e.g., through preserving the hypoxic TME) antitumor immune responses.

## Tumor-mediated lymphangiogenesis and immunosuppression

Besides blood vessels, the vascular network includes the lymphatic system. The lymphatic vessels (LVs) drain interstitial fluid consisting of a plethora of proteins, lipids, and cells from a tissue (for an extensive review, see ref. 36) to LNs. The (initial) blunt-ended capillaries that are embedded in the tissue have an intermittent basement membrane, discontinuous button-like cell–cell junctions, and the lack of pericytes and smooth muscle cells to facilitate interstitial fluid entry. These capillaries converge into precollecting vessels that traffic lymph to subcapsular sinuses (SCS) in LNs. Lymphatic endothelial cells (LECs) that line the SCS express CCL-21 and CCL-19 (T-cell chemoattractants) and CCL-1 (a DC chemoattractant). Guided through intranodal sinuses, DCs and T cells enter the T-cell zone (although the majority of T cells also enter directly from the blood via specialized vessels for lymphocyte trafficking that are found in secondary lymphoid organs such as LNs, called high endothelial venules or HEVs) that is a predominant site for DC–T cell interactions.

In the TME, cancer cell-derived ligands of VEGF receptor (VEGFR-)3 (VEGF-C, VEGF-D, and VEGF-A) can induce lymphangiogenesis, the equivalent of blood vessel angiogenesis that leads to the sprouting and attraction of LVs^37,38^. Tumor lymphangiogenesis leads to an expansion of the intratumoral and peripheral capillaries, collecting lymphatics, and draining lymph nodes (dLNs) and actively contributes to cancer cell dissemination^39^. Moreover, LECs function as antigen-presenting cells and induce immunological tolerance and promote the apoptosis of tumor-reactive CTLs^40^.

**Fig. 3:**
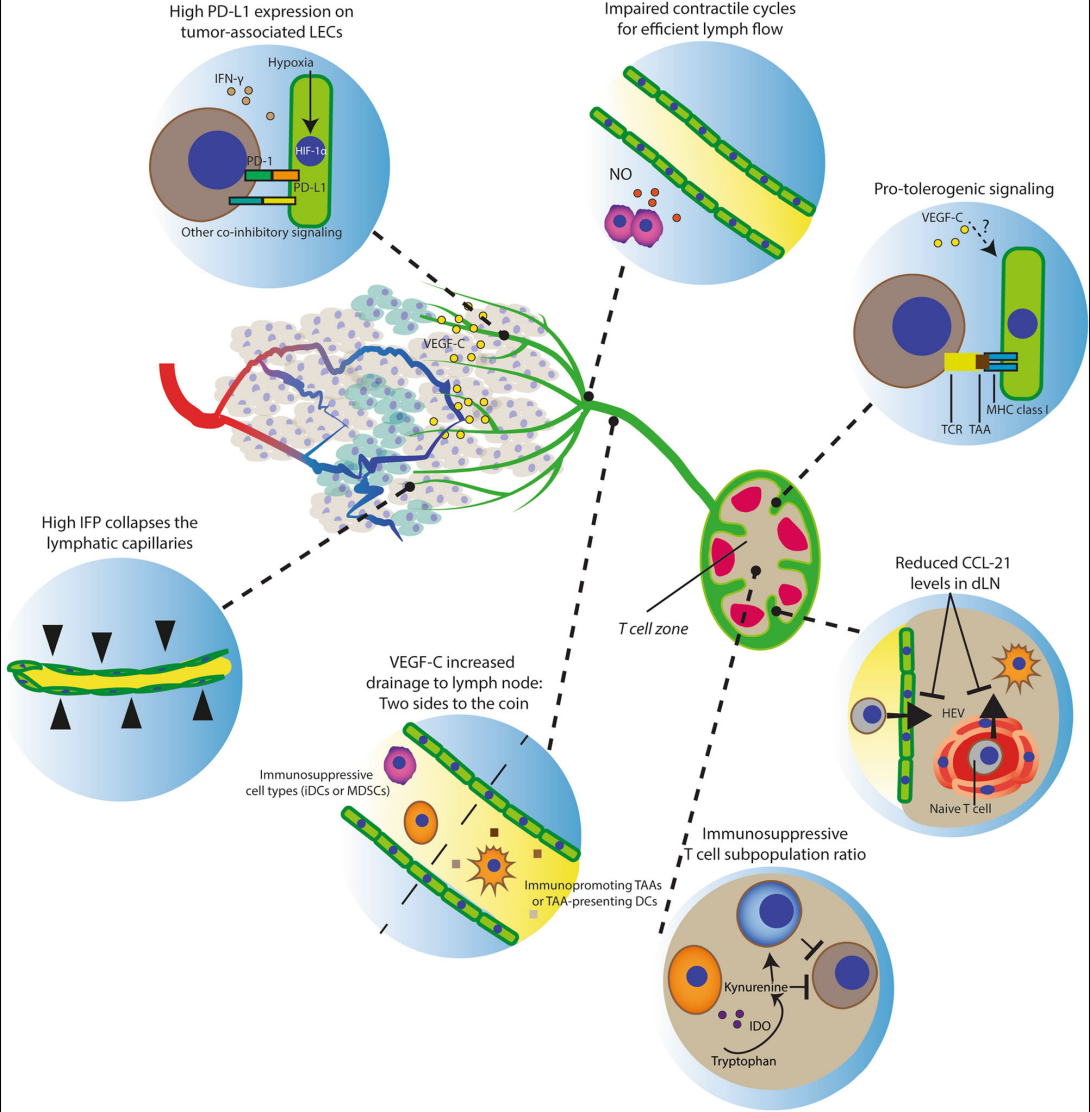
**The effects of tumor-associated lymphatic endothelium on antitumor immunity** The lymphatic vessels (green) guide antigens and DCs to lymph nodes to facilitate the DC–T cell interaction to prime T cells (only if the LN microenvironment allows this to be productive). Notably, lymphatic vessels are more common peritumorally, while intratumoral vessels are prone to collapse. Moreover, defects in contractile events for lymph flow impair drainage. Thus tumor drainage, albeit physically hampered in a tumor, is required for developing antitumor immunity. Importantly, additional LEC features (intrinsic or tumor induced) counteract the induction of an adaptive immune response. This is exemplified by the increased PD-L1 expression and protolerogenic cell surface protein composition (co-inhibitory over co-stimulatory factors). Drainage of immunosuppressive immune cell types (e.g., MDSCs, immature DCs) influence the LN microenvironment to favor immunosuppressive populations (e.g., T_reg_ and MDSCs) that facilitate lymphovascular premetastatic niche formation. Moreover, reduced CCL-21 levels in dLNs diminish the opportunity for DCs and naive T cells to interact and impairs T-cell retention for efficient expansion before LN egress.

Here we discuss some of the most salient features linking tumor-associated lymphatics to the regulation of antitumor immune responses in the TME, using melanoma as a paradigm of immunosuppressive and aggressive cancer harnessing the lymphatic system for dissemination (Fig. 3).

### Lymphatic capillaries in adaptive immune responses

A lymphatic score (based on the expression of lymphatic markers podoplanin, lymphatic vessel endothelial hyaluronic acid receptor (LYVE)-1, and VEGF-C) in metastatic cutaneous melanoma patient samples correlated positively with immune cell (CD45^+^) infiltration, including immunosuppressive subtypes (e.g., T_reg_ and inflammatory monocytes) and antitumor subtypes (e.g., CD8^+^ T lymphocytes). Consistently, when testing two independent mice models (including a transgenic K14-VEGFR-3-Ig model) that reduced peritumoral LYVE1-positive dermal lymphatic capillaries in a B16-F10 melanoma, general immune cell tumor infiltration declined including the number of T_reg_, inflammatory monocytes and CD8^+^ T lymphocytes. Moreover, this phenotype was associated with decreased DC trafficking from tumor to dLN. This indicates that, whereas lymphatic capillaries are required for T-cell infiltration to occur, they can also cause unproductive adaptive antitumor response^41^. Moreover, LECs can present self-antigens on MHC-I proteins to promote tolerance that is accentuated by secretion of immunosuppressive chemokines (TGF-β, IDO, nitric oxide (NO)), high PD-L1/L2 expression, and suboptimal co-stimulatory protein levels (CD80/CD86). Although LECs express basal levels of PD-L1 thereby modulating peripheral tolerance, PD-L1 expression is increased in tumor-associated LECs^42^, likely through HIF-1 or interferon (IFN)-γ, which are potent inducers of PD-L1/L2 expression in LECs. Thus, in case of successful tumoral infiltration by active CTLs, tumor-associated LECs may attenuate effector T cells’ cytolytic activity. Importantly, LECs increased PD-L1 expression when pulsed with a peptide of the model antigen ovalbumin (OVA). In the presence of PD-L1 blockade, co-culturing these LECs with OT-1 CD8^+^ cells resulted in improved cancer cell killing by OT-1 cells^42^, thus disclosing a LEC-mediated mechanism through which ICIs might stimulate CTL activity.

### Low lymphatic flow and high interstitial fluid pressure and immunosuppression

The lymphatic capillaries drain to larger contractile vessels referred to as collecting lymphatics that guide lymph toward dLNs. These regulate the lymphatic flow by contractions of surrounding smooth muscle cells. This is established by a spatiotemporally regulated NO production by LECs. Tumor-derived VEGF-C attracts LVs into the tumor (although it is predominant at the peritumoral regions^43^) and causes an increase in lymphatic pump activity (including contraction frequency that depended on VEGFR-3 activity, which causes tonic contraction)^44^. Thus VEGF-C can increase the tissue drainage of cells and TAAs. Additionally, MDSCs at the sites of inflammation are potent NO producers that impair lymphatic flow^45^; however, it is not clear to what extent this contributes in tumors. Still, cancer cells (especially when hypoxic) secrete cytokines and chemokines that recruit myeloid cells (a potential source of NO) and could thus impair these contractile cycles and the drainage of TAAs/TAA-presenting DCs to dLNs. Moreover, reduced lymph drainage and the lack of (functional) intratumoral LVs contributes to the high IFP^43^ and subsequent immunosuppressive effects. Improving the lymphatic vessel function as well as reducing the intratumoral MDSCs are seemingly important targets in improving antitumor immunity.

### The lymph node microenvironment and antitumor immunity

In essence, the LN is a tissue for the recognition and presentation of antigens to prime or tolerogenize adaptive immune responses. A tumor drains various secreted factors that influence the LN microenvironment in favor of immunosuppression. This counteracts antitumor immunity and generates a hospitable environment for the seeding and growth of cancer cells (“lymphovascular premetastatic niche”). In line with this, B16-F10 cells injected into the LN, but not subcutaneously, are rejected in a CD8^+^ cell-dependent manner^46^. Moreover, micrometastasis-free dLNs from melanoma patients have increased levels of certain immunostimulating cytokines as compared to non-sentinel LNs and micrometastases positive LNs. These include IFN-γ (suggesting TAAs-specific immunity), IL-2 (B and T-cell proliferation stimulus), and granulocyte macrophage colony-stimulating factor (DC maturation factor)^47^. Thus, initially, an immune response is incited in a dLN that can also be sufficient to prevent colonization. Yet the tumor eventually overcomes this protective effect. In agreement with this, in the presence of a subcutaneous B16-F10 tumor, intralymphatic B16-F10 injection resulted in effective tumor growth^46^. This can be a result of tumor-derived secreted factors and immature DCs and recruitment of MDSCs. Regarding the former study using an OVA-expressing B16-F10 melanoma model, additional VEGF-C overexpression led to reduced IFN-γ-producing CD8a^+^ OT-1 cells in the dLN, possibly due to enhanced lymph flow and LEC-mediated tolerogenic events^48^. Moreover, in a different melanoma model (B16-F1), CCL-21 expression in dLNs reduced progressively in time after tumor injection, as compared to unchallenged LNs^49^. This could possibly result in an impaired T-cell retention, which enables clonal expansion before LN egress^50^.

Thus the lymphatic system can support (draining of TAAs/TAA-presenting DCs) as well as attenuate (tolerogenic events) antitumor immune responses.

## Vascular targeting approaches: limitations and opportunities for immunotherapy

The discussion so far establishes that the tumor vasculature (both ECs and LECs) is an essential regulator of the intersection between cancer cells and immune compartment within the TME. By extension, tumor vasculature can henceforth play an important role in regulating responses to cancer immunotherapy^28^. Briefly, immunotherapy aims to modulate the host’s immune system to promote antitumor immunity and it broadly includes treatments with cytokines/immunomodulatory drugs, monoclonal antibodies (mAbs), adoptive cell transfer, and anticancer vaccines, such as DC vaccines^51–53^. However, the current landscape of cancer immunotherapy is largely dictated by ICIs, principally because of their clinical success and prominent and durable responses in patients of several histological tumor types^54–56^. The most prominent ICIs are mAbs blocking the activity of CTLA-4, PD-1, and PD-L1. Emerging evidence, moreover, highlights that the type of cancer cell death may favor or impede antitumor immunity and regulate the success of ICIs in combinatorial regimens^57^. Indeed, antitumor immunity can be accentuated via the induction of immunogenic cell death (ICD) in cancer cells, thus acting as ‘in situ’ vaccines^58,59^. Major hallmarks of ICD are the ER stress-regulated and spatiotemporally defined emission of danger signals (most prominently, surface calreticulin, secreted ATP, and passively released high mobility group box-1, nucleic acids, dsRNA, dsDNA)^60,61^. Moreover, ICD is uniquely associated with ‘altered-self mimicry’ elicited by type I IFN cytokines (consisting of IFN-α and IFN-β) and a pathogen response-like chemokine signature (consisting of C-X-C ligand (CXCL)-1, CCL-2, CXCL-10, or homologs thereof)^62,63^. Of note, cancer cells succumbing to ICD can also be used for creating next-generation DC-based vaccines^64^.

**Fig. 4:**
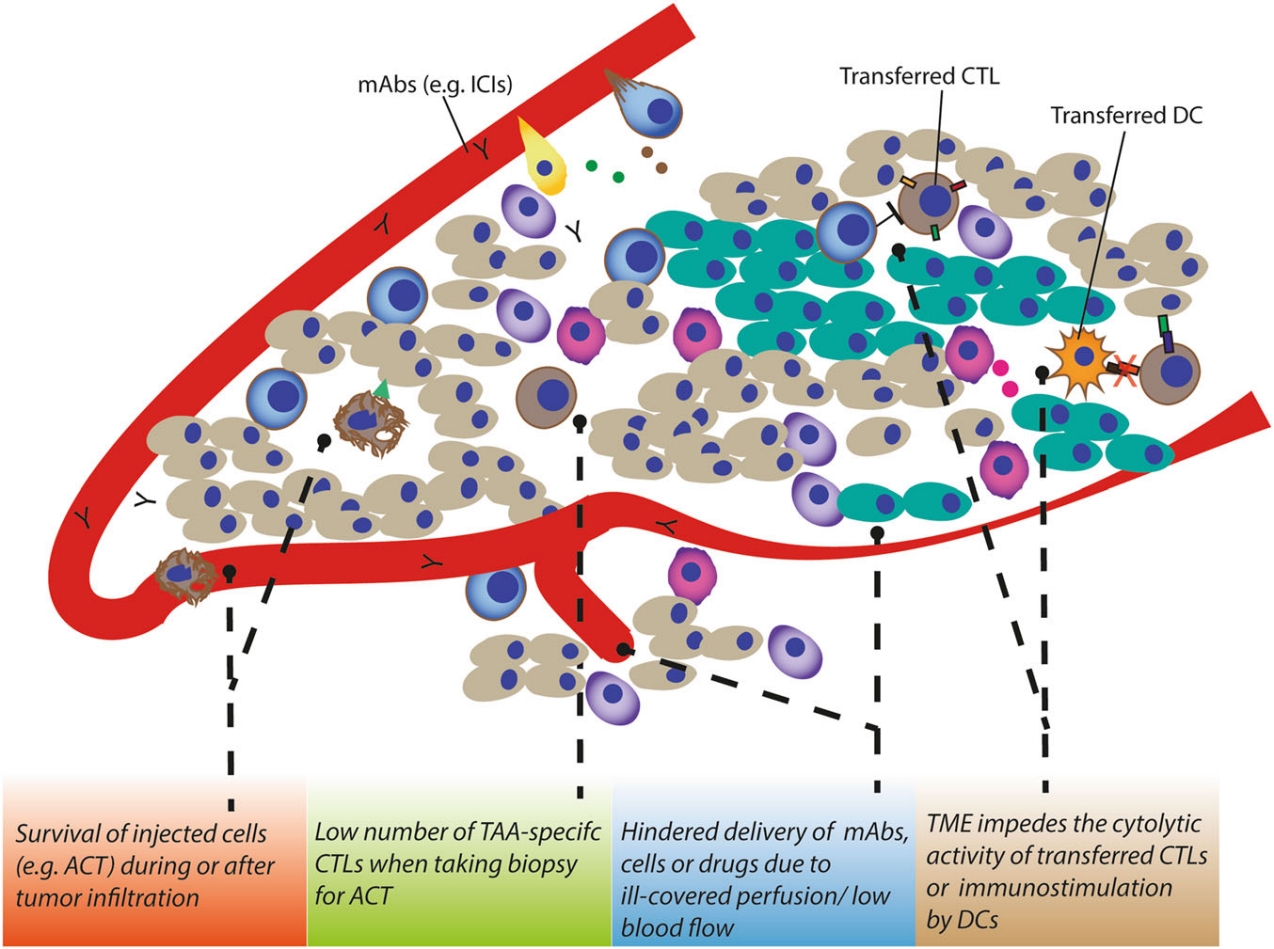
**Hurdles established by the tumor vasculature** that limit immunotherapy efficacy As discussed above, the TME often thwart CTL presence in the TME due to inducing apoptosis/ill-adhesion or by functional inhibition even when infiltrated. This low number of TAA-specific CTLs affects the harvest from tumor biopsies for adoptive T-cell transfer-based immunotherapy. Moreover, delivery of administered regimens including monoclonal antibodies, DCs, and T cells can be hindered due to ill-perfusion. Yet, the TME can still functionally inhibit the transferred DCs when infiltrated

Although immunotherapy has prolonged survival of many cancer patients (as evidenced by a string of Food and Drug Administration approvals in a relatively short span of time), there are still various hurdles limiting its therapeutic efficacy^65,66^. These are in large part caused by the profoundly immunosuppressive TME and cancer cell-autonomous mechanisms of immunoevasion (e.g., loss of TAAs or MHC expression levels, dysregulation of IFN signaling, dysregulation of danger signaling, immunogenic phagocytosis) (reviewed in refs. 66,67). As discussed above, the aberrant tumor vasculature can counteract immunotherapy due to ill-delivery of the mAbs (as a result of the immature and badly structured blood vasculature) and restrain anticancer immune responses by favoring the presence of immunosuppressive immune cells (e.g., presence of MDSCs, M2 TAMs, and T_reg_ cells (Fig. 4) over immunostimulatory immune cells (mainly CD8^+^ T cells). Compelling evidence indicate that spatial, functional orientation and density of T lymphocytes within the tumor is associated with good patient prognosis across many cancer types^64,68,69^.

Based on these emerging lines of evidences, we surmise that targeting of tumor vasculature might improve the efficacy of cancer immunotherapy. In fact, this is one of the main reasons behind recent proposals to target the tumor vasculature in combination with cancer immunotherapy. In the next section, we describe and discuss some potential combinatorial strategies using antiangiogenic and immunotherapy approaches.

### Antiangiogenic treatment, vessel normalization, and immunotherapy

Targeting of the VEGF/VEGFR axis has been the most preferred combinatorial approach for immunotherapy-related studies. Initially, monotherapy with antiangiogenic agents, such as the anti-VEGF antibody bevacizumab, by blocking the VEGF/VEGFR-dependent survival and growth of the blood vasculature, was thought to starve the tumor thus halting tumor progression and improving patient survival. In spite of promising initial preclinical results, this vessel-targeting therapy, called vessel blocking, did not elicit the expected results in cancer patients and failed to show substantial improvements in response rates or survival benefits^70^. Later on, preclinical studies showed that vessel pruning leads to an increase hypoxic (but not ischemic) tumor areas^71^, which in turn supported tumor growth and metastatic dissemination. Indeed, hypoxia induced by anti-VEGF/VEGFR therapy may be in part responsible for the angiogenic relapse and therapy resistance observed after vessel blocking strategies, which may involve distinct immunosuppressive immune-cell populations, including Gr1^+^CD11b^+^ and TAMs^72^. A recent study showed that these myeloid cells are recruited by the cancer cell-derived, angiostatic chemokine, CXCL14, which instigated PI3K signaling in these myeloid cells. In line, inhibition of this pathway was required for the durable effects of antiangiogenic therapy^21^.

However, in experimental mouse models, Bevacizumab (Avastin, a recombinant humanized antibody that binds VEGF isoforms) treatment resulted in a transient remodeling of the tumor vasculature by increasing the number of matured (i.e., pericyte covered) vessels, decrease permeability, reduce IFP, and increase perfusion in neuroblastoma xenografts. This vessel ‘normalizing’ effect was transient as the observed intratumoral penetration of topotecan and etoposide only improved the first days after Bevacuzimab treatment^73^. Another study showed that DC-101 (mouse VEGFR-2 specific monoclonal antibody) treatment of glioma xenografts increased vessel normalization that is associated with a time window for the synergistic effect of the combined treatment with radiotherapy, an inducer of ICD^71^.

Hence, these data suggest a window of opportunity to establish a synergistic effect between tumor vessel-normalizing agents and immunotherapy^74^.

They also suggest that ‘normalizing’ or ‘healing’, rather than destroying, the erratic tumor vasculature may restore normal structural functional aspects of the vessels, which elicit a better therapeutic outcome. In line with this, ‘vessel normalization’ by improving vessel functionality results in better perfusion of the tumors and by increasing the transporting capability of vessels improves both drug delivery (of small chemotherapeutics as well as mAbs) and therapy responses, which strongly depend on adequate tumor blood supply^75,76^. Moreover, the resulting improvement in tumor oxygenation may increase the efficacy of immunotherapy. Indeed, as mentioned above, hypoxia and poor intratumoral infiltration of T cells, caused by the poor perfusion of the aberrant tumor vessels, attenuate the efficacy of ICI-based immunotherapy^32,77^. Opposite to this, hyperoxygenation increases CTL activity and correlates with improved clinical responses to ICIs^78^.

In the context of immunotherapy, DC-101 treatment also associated with an increased B16-F10 melanoma infiltration of adoptively transferred T cells and an enhanced tumor growth delay^79^. In addition, DC-101 treatment led to tumor vessel normalization and reduced tumor hypoxia only in low (10–20 mg/kg) but not high dose (40 mg/kg) treatments. Moreover, this was accompanied with important changes in the TME with a shift toward tumor-suppressing Th1-mediated immune responses, including TAM polarization to an M1-like phenotype and increased CD4^+^ and CD8^+^ T-cell tumor infiltration. These changes in the TME also enhanced the effect of vaccine-based immunotherapy^80^. In addition, transient targeting of VEGF/VEGFR axis may reverse DC maturation defects^81^ and lower VEGF-A induced PD-1, TIM3, and CTLA-4 expression on CD8^+^ T cells^34^; however, Bevacizumab may also inhibit the phagocytic ability of DCs and macrophages^82^.

Besides blocking the VEGF/VEGFR axis, other strategies have been shown to induce vessel normalization^83,84^. Recently, the antimalarial compound and first-generation autophagy inhibitor chloroquine (CQ) was found to elicit in vivo vessel normalization through the activation of the Notch-signaling pathway in blood ECs^85^, leading to a more quiescent EC phenotype^86^. Both the tumor vasculature-normalizing and antimetastatic effects of CQ were completely blunted when melanoma cells were implanted in mice lacking *Notch1* in ECs. By normalizing the abnormal tumor vasculature, CQ attenuated tumor hypoxia and caused the generation of a more EC solid barrier that impeded cancer cells’ intravasation and metastasis^86^. Intriguingly, a recent preclinical study showed that, in spite of its mild immunosuppressant effects, CQ does not impair antitumor immunity in vivo and can synergize with immunotherapy^87,88^. Another therapeutic strategy may entail reprogramming the ECs' glycolytic phenotype. Recent studies revealed that ECs depend predominantly on glycolysis for ATP production. Furthermore, this glycolytic phenotype is aggravated in the TME by the enhanced VEGF signaling and contributes to vascular dysfunction^89,90^.

A recent study showed that blockade of the key glycolytic activator 6-phosphofructo-2-kinase/fructose-2,6-bisphosphatase 3 normalized blood vessels, an effect that was associated with a tightened vascular barrier (fewer metastases) and increased perfusion (improved chemotherapy efficacy)^91^. Thus pharmacological inhibitors targeting EC glycolytic metabolism could reverse tumor-induced alterations in ECs leading to a vessel normalization phenotype^92^, a therapeutic strategy warranting further experimental and clinical confirmation validation.

Moreover, recent insights show the relevance of non-protein-coding micro-RNAs (miRNAs) in angiogenesis (see refs. 93,94 for a more detailed overview) by regulating gene expression via RNA interference. For example, pro-angiogenic miRNAs can be induced by hypoxia (including miR-210 and miR-494)^95^ or, oppositely, certain miRNAs affect the VEGF/VEGFR pathway (e.g., miR-16 (that also interferes with TGF-β signaling))^96^ to modulate angiogenesis. Interestingly, cancer cell-secreted vesicles containing miR-494 can promote angiogenesis in ECs^97^. Thus, as tumor-associated conditions can promote the expression of miRNAs to support the highly angiogenic TME (either cell autonomously or via cross-communication), miRNAs could be considered as potential targets of antiangiogenic/vessel-normalizing approaches. Nevertheless, this is still an emerging field that requires further research to reach a better understanding of how (specific) targeting miRNAs may enhance immunotherapy efficacy.

Aside from VEGF-A, other proteins that promote immunosuppression and angiogenesis may be interesting targets. IDO can stimulate angiogenic events (effect of kynurenine on ECs^98^) besides establishing immunosuppressive events (tryptophan depletion). Interestingly, in the context of immunotherapy, IDO inhibition synergizes with ICI approaches in preclinical models^99^, which may therefore be contributed through IDO-mediated effects on tumor vasculature. Furthermore, secretion of galectin-3 (whose expression is induced by hypoxia and nutrient deprivation) inhibits the effector function of CD8^+^ T cells^100^ and also invigorates VEGF and bFGF-induced angiogenic events in ECs^101^. Therefore, targeting these crosstalks and signaling axis could shape a TME in favor of antitumor immunity; however, these possibilities need further investigations and validation in preclinical models.

Interestingly, although vessel normalization can result in improved lymphocyte infiltration and a less therapy-resistant TME, the infiltration of CD4^+^ T cells can induce vessel normalization as well. In a recent and elegant study, adoptive CD4^+^ T-cell transfer was associated with reduced hypoxia and leakiness, and increased perfusion, while CD4 depletion reduced vessel pericyte coverage^102^. Together this suggests a reciprocal feedback loop in which a lymphocyte-admissible TME by vessel normalization has subsequent positive effects on the vasculature integrity.

Despite only few studies focusing on targeting the tumor-associated lymphatic structures, its relevance for immunotherapy outcome should not be underestimated. A study utilizing a B16-F10-OVA model showed that VEGF-C overexpression was able to protect against the antitumor immune response elicited by OVA vaccination^48^. In a transgenic model removing dermal lymphatic capillaries, the efficacy of a vaccination approach was impaired (due to impaired development of antigen-specific CD8^+^ cells), whereas an ACT approach (OT-1 cells activated with OVA-peptide-loaded DCs) was more effective (possibly due to reduced immunosuppressive TME)^41^.

Taken together, the aforementioned studies suggest that targeting angiogenesis, with vessel-normalizing strategies in particular, can improve the efficacy of immunotherapies.

### Specific tumor vasculature targeting strategies to improve outcome of anticancer therapy

Administration of antitumor immunity-stimulating cytokines such as IL-2, TNF-α, and IFN-γ can be beneficial; however, it is limited by maximum tolerated doses in patients^103^. New approaches have been developed to restrict the dose required for a beneficial therapeutic effect on the tumor. Treatment of colorectal cancer-bearing mice with TNF-α or IFN-γ conjugated to the tumor vascular homing peptide TCP-1 resulted in tumor growth delay, increased TUNEL (terminal deoxinucleotidyl transferase-mediated dUTP-fluorescein nick end labeling) staining in the tumor, and reduced systemic toxicity compared to unconjugated cytokines. Importantly, the TME also improved as the CD8^+^ (TCP-1/TNF-α) and CD4^+^ (TCP-1/TNF-α, TCP-1/IFN-γ) cell infiltration increased^104^ and the vasculature normalized (TCP-1/TNF-α)^105^. TCP-1/TNF-α improved 5-FU delivery and, due to the synergistic effects, improved drug-induced tumor control^105^. In addition, conjugating TNF-α to a Cys-Asn-Gly-Arg-Cys (NGR) peptide (recognizing an aminopeptidase N (CD13) isoform on tumor-associated ECs) led to increased adhesion molecule expression on ECs, increased CD8^+^ T cell infiltration in B16-OVA melanoma, and improved outcome of both ACT (with OVA-specific in vitro*-*activated OT-1 cells) and DC-OVA vaccine approaches^106^. Other approaches use a small immune protein (L19) to target the additional extra-domain (ED-B) of fibronectin associated with tumor neovasculature. Combined with either dacarbazine^107^ or radiotherapy^108,109^, L19-IL2 treatment enhanced the efficacy of the therapy modality, which was suggested to be CD8^+^ T-cell dependent^108^ possibly due to the ICD-inducing ability of radiotherapy that enhances the CD8^+^-mediated immune response.

## Conclusions

To maintain a cancer cell permissive and immunosuppressive microenvironment enabling tumor growth and dissemination, cancer cells educate and corrupt stromal cells. Emerging evidence indicate that cancer cell-induced effects on both the blood and lymph endothelium are crucially involved in the generation and maintenance of an immunosuppressive TME. In particular, the tumor vasculature can actively suppress antitumor immune responses by providing a barrier to T cells' infiltration in the tumor, by selectively killing T cells, or by increasing tolerogenicity against TAAs. Given that spatial, functional orientation and density of T lymphocytes within the tumor (i.e., the immunoscore^110^) is one of the main predictor of therapy responses in patients, this has generated the therapeutic perspective of targeting the abnormal tumor vasculature to relieve critical TME-associated conditions that antagonize the efficacy of immunotherapy. In line with this, an increasing amount of preclinical data indicate that vessel-normalization strategies, eliciting a transient improvement of the aberrant structural and functional features of the tumor blood vessels, results in lowering tumor hypoxia and increasing drug delivery, thereby enabling immune cell infiltration and synergize with immunotherapies for more durable effects^111^. These findings have important implications for the design of a combinatorial strategy using vessel-normalizing agents with immunotherapy. However, there are many outstanding questions and challenges that remain to be addressed.

First, alternative strategies to VEGFR blockade aiming to sustain the effects of antiangiogenic therapies are required. To this end, emerging ECs metabolic signatures and EC trafficking pathways may offer more efficient alternative targets and the availability of pharmacological inhibitors of these pathways (e.g., CQ) should favor their future applications. Also, the role of other stromal cells, like cancer-associated fibroblast should be considered as these can promote angiogenesis as well. Given the emerging relevance of the dynamic intersection between the immune cells (i.e., T cells, myeloid cells, DCs) and ECs, in angiogenesis, and relapse after antiangiogenic therapy, more studies are needed to reveal potential targets that blunt the recruitment of immunosuppressive immune cells fostering tumor regrowth. Further, when applying tumor vasculature targeting regimens, the effects of additional modulation of the lymphatics (by, e.g., VEGFR-3 inhibition) should be carefully considered, since whether modulation of lymphangiogenesis overcomes tolerogenic events or impairs stimulation of an adaptive response remains still ill-defined. Moreover, whether the concept of vessel normalization can be extended to lymphatic vessels is still elusive.

In conclusion, targeting the tumor vasculature to induce vessel normalization may provide a promising strategy to optimize the efficacy of currently employed immunotherapies as it could lower the level of immunosuppression in the TME. Yet, it is clear that, if we want to exploit the full potential of the immune system to cure cancer, we will have to act at multiple levels in order to ‘normalize’ the TME.
